# Molecular and Serological Detection of *Mycobacterium tuberculosis* in Dairy Cattle From Sylhet, Bangladesh: Implications for Zoonotic Transmission

**DOI:** 10.1155/2024/3653654

**Published:** 2024-10-30

**Authors:** Md. Mukter Hossain, Md. Masudur Rahman, Md. Mahfujur Rahman, Hemayet Hossain, Ruhena Begum, Md. Shahidur Rahman Chowdhury, Md. Rafiqul Islam, Md Bashir Uddin

**Affiliations:** ^1^Department of Medicine, Faculty of Veterinary, Animal and Biomedical Sciences, Sylhet Agricultural University, Sylhet 3100, Bangladesh; ^2^Department of Pathology, Faculty of Veterinary, Animal and Biomedical Sciences, Sylhet Agricultural University, Sylhet 3100, Bangladesh; ^3^Department of Anatomy and Histology, Sylhet Agricultural University, Sylhet 3100, Bangladesh

**Keywords:** cattle, *Mycobacterium tuberculosis*, PCR, serum ELISA, zoonosis

## Abstract

This study aimed to determine the seroprevalence and molecular detection of *Mycobacterium tuberculosis* (*M. tuberculosis*) in dairy cattle using ELISA and PCR techniques. A total of 500 samples (250 blood and 250 milk) were collected from various farms in Sylhet, Bangladesh. The seroprevalence was found to be 5.6% in blood samples, with PCR confirming 1.60% and 2.80% positivity in blood and milk samples, respectively. These findings highlight the zoonotic potential and public health significance of *M. tuberculosis* in cattle, suggesting a need for integrated One Health surveillance.

## 1. Introduction

*Mycobacterium tuberculosis* (*M. tuberculosis*), the causative agent of tuberculosis (TB), is a significant zoonotic pathogen affecting both humans and animals. While primarily infecting humans, *M. tuberculosis* can also infect cattle, posing a risk for zoonotic transmission. This study is the first to report the prevalence of *M. tuberculosis* in dairy cattle in Sylhet, Bangladesh, highlighting the need for integrated surveillance to mitigate public health risks. Other members of the *Mycobacterium tuberculosis* complex (MTBC) comprising *Mycobacterium bovis, M. microti, M. caprae, M. africanum, M. canettii,* and *M. pinnipedii*, which are etiological agents of mammalian TB [1]. *M. tuberculosis* and *M. bovis* are the major causes of TB, highly pathogenic, and capable of infecting many animal species, and thus likely to be the source of tuberculous infection in humans. The highest prevalence of human TB is found in Asia, where China, India, Bangladesh, Indonesia, and Pakistan collectively make up over 50% of the global burden [2]. TB has no geographical limitations and is found in most underdeveloped countries with insufficient or nonexistent surveillance control measures [3]. Humans are considered as the principal reservoir hosts for *M. tuberculosis*. Transmission by direct contact or droplet transmission is also possible among high-risk people, such as veterinarians and animal keepers, who are in frequent contact with animals [4]. Although human-to-cattle transmission of *M. tuberculosis* has been reported [5], the isolation of *M. tuberculosis* from any species other than humans, especially cattle, is interesting and important. *M. tuberculosis* causes less severe disease in cattle than *M. bovis* infection. It is imperative to determine that the source of the pathogen is cattle as there is a possibility of cross-contamination of samples [6]. It is spread through the consumption of contaminated feed and water, feces, or exudates from infected animals that contain tubercle bacilli. Although the bovine tubercle bacillus is the cause of bovine tuberculosis (bTB) in cattle, it is still commonly used to refer to bovine tubercle bacillus strains regardless of the host. The disease has also been reported in goats, sheep, pigs, horses, cats, and dogs, as well as the fennec fox, bison, buffalo, badger, wild and feral pigs, antelope, camels, and humans and nonhuman primates [1]. The strongest predictor of disease occurrence is cattle movements, particularly those from locations where bTB is recorded [7].

*Mycobacterium bovis*-caused bTB is a significant zoonotic disease in dairy cattle in Bangladesh exhibiting a seroprevalence of 7.5% [8]. The potential identified risk factors include cattle source, farm population size, and the density of stocking linked to this condition. Detection of TB in individuals with occupational exposure to cattle in Bangladesh revealed cases caused by *M. tuberculosis* but not *M. bovis* [9]. Diagnostic protocols for TB-infected cattle entail a blend of polymerase chain reaction (PCR) assays targeting MTBC and *M. bovis*–specific molecular and serological detection. For example, in Assam, molecular detection of *Mycobacterium* in dairy cattle revealed positive reactors through tuberculin test, the isolation of *Mycobacterium* from tissue samples, and successful amplification of *Mycobacteria* genus–specific genes [10]. These findings underscore the importance of molecular and serological detection methods in understanding and preventing zoonotic transmission of *M. tuberculosis* in dairy cattle.

In the current study, we document the *M. tuberculosis* infection of cattle from different farms and various locations in the Sylhet region of Bangladesh. The majority of cases of *M. tuberculosis* in cattle occur in countries with high rates of human infection, and this is probably where the disease was initially introduced into cattle populations [11]. Bangladesh is among the top 30 countries with a high TB burden, accounting for 3.6% of all cases worldwide. According to WHO estimates from 2020, Bangladesh has an incidence and mortality rate of 221 cases and 24 deaths per 100,000 people, respectively, from TB. So far, very few prevalence studies of *M. tuberculosis* infections in cattle have been published to date, despite the fact that human infections are a concern in our country. Therefore, the goal of this research was to determine the seroprevalence and molecular detection of *M. tuberculosis* for evaluating bovine samples in order to avoid zoonotic transmission at the cattle–human interface.

## 2. Materials and Methods

### 2.1. Study Area

**Figure 1:**
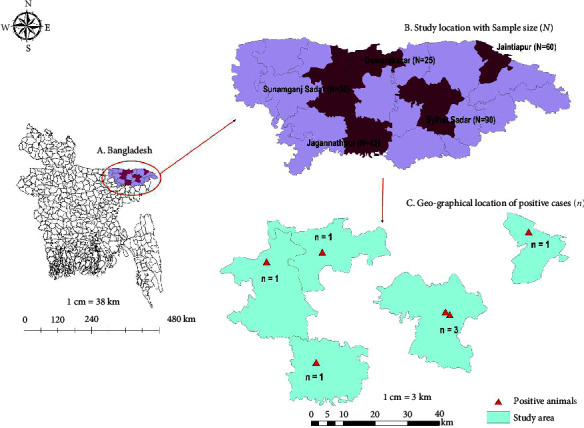
The geospatial map showing the study area with sample size and geographical coordinates of each PCR positive (milk sample) cases. The map was created with ArcMap 10.7 (ESRI, USA) software.

This study was conducted in the Sylhet region of Bangladesh. Blood and milk samples of crossbred dairy cattle were collected from different intensive commercial and free-grazing cattle in Sylhet division (Sylhet and Sunamganj district) of Bangladesh (Figure 1).

### 2.2. Collection of Samples

A total of 500 samples (250 milk samples and 250 blood samples) were collected. Milk samples were collected aseptically directly in the Falcon tube from the udder of cattle. A 10-mL blood sample was taken from the jugular vein after the animal had been handled and restrained properly. Five milliliters (5 mL) of blood was collected and kept in the EDTA-containing vacutainer (BD Biosciences, Franklin Lakes, New Jersey, USA). Remaining 5 mL of blood was transferred to a vacutainer tube (without EDTA; BD Biosciences, Franklin Lakes, New Jersey, USA) and left at room temperature for 15–30 min to clot.

### 2.3. Serum Separation From Blood Samples

For serum separation, the blood clot was removed by centrifugation in a refrigerated centrifuge at speeds of 12, 000 × G for 15 min. The resulting supernatant was designated as serum. After centrifugation, the clear supernatant was transferred into a polypropylene tube. The serum samples were then stored at −20°C for further analysis.

### 2.4. Serological Examination of *M. tuberculosis*

Each sample was tested in duplicate through enzyme-linked immunosorbent assay (ELISA). Optical densities were then measured at 450 nm using a Multiskan FC (Thermo Fisher Scientific). A total of 250 serum samples from cattle of different farms in Sylhet, Bangladesh, were analyzed using the ELISA. An indirect ELISA was carried out using the Bovine TB Antibody ELISA Kit (Cat. No. SL0127Bo, Sunlong Biotech Co. Ltd., Hangzhou, Zhejiang, China) for the qualitative detection of *M. tuberculosis* antibodies in serum. The kit includes a microplate with precoated wells containing bTB antigen. The results were expressed using an ELISA index (EI), which is the ratio between the mean optical density (OD) of the positive control and the mean OD value of the sample.

### 2.5. DNA Extraction From Blood Samples

The phenol, chloroform, and isoamyl alcohol techniques were used to extract genomic DNA. In brief, the blood samples were homogenized by repeatedly inverting the tubes, and 400 *μ*L of blood samples was added to an Eppendorf tube containing 700 *μ*L of deionized water. The mixture was then centrifuged at 10,000 rpm for 10 min. After discarding the supernatant, again 700 *μ*L of deionized distilled water was added and homogenized the mixture using a vortex machine. Subsequently, the mixture was centrifuged at 10,000 rpm for 10 min, and then, supernatant was discarded. Furthermore, 200 *μ*L lysis buffer and 2 *μ*L proteinase *k* were added to the tubes, homogenized by inverting the tube multiple times, and then incubated overnight at 37°C. After incubation, 100 *μ*L of 4.5 M sodium chloride (NaCl) was added to the tubes, which were then homogenized by inverting several times. The tubes were then filled with 225 *μ*L of chloroform and shaken for 10 min. The mixture was then centrifuged at 14,000 rpm for 10 min. The tube had three layers, and therefore, around 200 *μ*L of the aqueous phase/upper phase was moved into another sterile Eppendorf tube. The 200 *μ*L of isopropanol was poured into the tube and stirred with a vortex or by inverting it numerous times. The samples were centrifuged at 14,000 rpm for 15 min. After removing the supernatant, 500 *μ*L of 70% ice-cold ethanol was added to the tube. The tube was kept at room temperature for 15 min and then centrifuged again at 14,000 rpm for 15 min. Removed the supernatant and dried the tube containing the particle for 10 min at room temperature. After that, the DNA pellet was resuspended in 100 *μ*L of TE buffer (1%). The tube was then incubated overnight at 37°C. Pipetting the extracted DNA for homogenization, the isolated DNA was stored at −20°C for further analysis.

### 2.6. Extraction of Bacterial Genomic DNA From Milk Samples

The DNA extraction from milk sample was performed according to the manufacturer's instructions (Monarch® Genomic DNA Purification Kit, UK). Briefly, 20 *μ*L of proteinase K and 200 *μ*L of lysis buffer were added to 200 *μ*L of the sample solution and incubated at 56°C for 10 min. Following the incubation step, a total volume of 200 *μ*L of absolute ethanol was added to the lysate. After that, the sample was washed and centrifuged in accordance with the manufacturer's protocol. The elution buffer provided in kit was used to elute the nucleic acid. Finally, the DNA was eluted using 50 *μ*L elution buffer and stored at −20°C until further use.

### 2.7. PCR Amplification

PCR was performed using a thermal cycler (DLAB Scientific Inc.) with the following cycling conditions: initial denaturation at 94°C for 5 min, followed by 35 cycles of denaturation at 94°C for 30 s, annealing at 62°C for 30 s, and extension at 72°C for 1 min, with a final extension at 72°C for 10 min. The target sequence (369 bp) was amplified using the previously described [12] primers pair (forward: 5′-ATGTGCGAGCTGAGCGATG-3′) and (reverse: 5′-AAAGGAGCACCATCGTCCAC-3) on the isolated bacterial DNA. Primers were utilized in a final reaction volume of 20 *μ*L using 10 *μ*L of master mix (2x conc. with UDG). Each reaction contained 5 *μ*L of extracted DNA, 10 *μ*L of master mix, and 5 *μ*L of primers (forward and reverse). The mixture was thoroughly mixed by repeatedly pipetting it in a PCR tube. The PCR product was then identified by electrophoresis visualizing the *M. tuberculosis*–specific band at 369 base pairs (bp). A 100-bp ladder (AddBio Inc., Korea) was included to measure the PCR product size estimation.

### 2.8. Statistical Analysis

The data from the laboratory were gathered and organized into the Microsoft Excel 2013 (USA) spreadsheet. Subsequently, both the parametric and nonparametric data were coded, sorted, and thoroughly checked to minimize errors. Following this, the data were analyzed for Chi-square (*χ*^2^) test using R and RStudio (Version 4.2.1). Prevalence was estimated using the following formula:

(1) $$\begin{array}{c} \text{Prevalence}\left(\%\right)=\frac{\text{The portion of infected individual}}{\text{Total sampled individuals}}\times 100. \end{array}$$

The precision of this estimate was ensured with a 95% confidence interval (CI). The prevalence, along with the corresponding CI, was calculated precisely using the binomial exact test method. For the univariate analysis, all relevant predictor variables associated with the response variable were considered. In instances where more than 20% of the cells had an expected count of less than 5 during the Chi-square (*χ*^2^) test, we opted for Fisher's exact test instead of the standard univariate Chi-square (*χ*^2^) test. To visualize the geospatial location of the study, a different map was created. The sample size dot map and choropleth map for prevalence were constructed using ArcMap 10.7 (ESRI, USA).

## 3. Results

### 3.1. Seroprevalence of *M. tuberculosis*

All blood samples underwent serological assay using the *Mycobacterium* antibody (*Mycobacterium*-Ab) ELISA Kit (Sunlong Biotech®, China). The overall seroprevalence of *M. tuberculosis* in blood samples from Sylhet and Sunamganj district was estimated to be 5.6% (14/250). Among intensive commercial dairy farms, the seropositive ratio for antibodies to *M. tuberculosis* was 5.33% (8/150), which was slightly lower than cattle from free grazing (6.0%; 6/100). In addition, 2.0% (5/250) of dairy cattle showed serological suspicions of *M. tuberculosis* (Table 1). Geographically, the highest prevalence of antibodies to *M. tuberculosis* was found in serum samples from the Dowarabazar Upazila of Sunamganj district (12.0%; CI: 2.55–31.22) followed by Farm 4 and Farm 5 (10.0%), Farm 1 (7.5%; CI: 1.57–20.39), Sunamganj Sadar (6.67%; CI: 0.82–22.07), and Farm 2 (5.0%; CI: 0.13–24.87) (Table 1). Sera from Farm 3 and Farm 6 did not contain any antibodies (Table 1). Nevertheless, there was no statistically significant (*p* > 0.05) variation in the occurrences of *M. tuberculosis* between the regions.

### 3.2. Molecular Detection of *M. tuberculosis*

**Figure 2:**
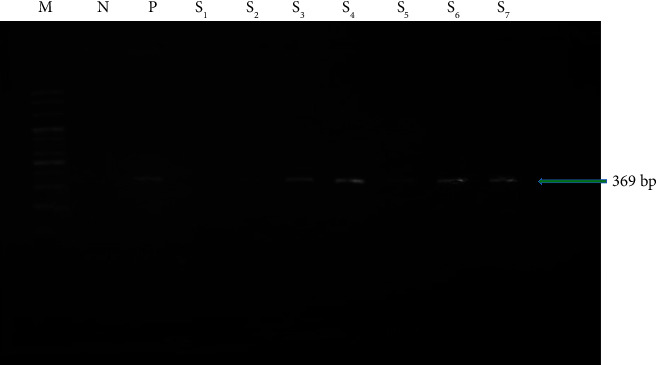
PCR amplification of seropositive *Mycobacterium tuberculosis* (Lane; M = marker DNA, N = control −ve, P = control +ve, Lane-S_1_–S_7_ samples).

For the confirmation, *M. tuberculosis*–specific nucleotides were used for PCR. Both milk and blood samples were molecularly analyzed to detect *M. tuberculosis* (Figure 2). In the case of blood samples, 4 out of 250 samples (1.60%; CI: 0.13–24.87) tested positive for PCR (Table 2). On the other hand, 7 out of 250 milk samples (2.8%; CI: 1.13–5.68) were discovered to be positive (Table 3), which was relatively higher than the blood samples (1.60%). Among the blood samples, a higher rate of *M. tuberculosis* (5.0%) was detected in cattle from Farm 4, while Farms 3 to 7 had no positive blood samples (Table 2). Furthermore, *M. tuberculosis* was detected more frequently in the milk of cattle sampled from Dowarabazar Upazila of Sunamganj district (8.0%; CI: 0.98–26.03), followed by Farms 1, 2, and 7 of Sylhet district at 5.0% (Table 3).

The univariate analysis (Table 4) of different explanatory variables associated with the detection of *M. tuberculosis* in dairy cattle revealed that the samples from free-grazing animals had a higher occurrence (6.0%; CI: 2.23–12.60, OR: 1.13) compared to cattle from intensive commercial dairy farms (5.53%; CI: 2.33–10.24). Similarly, the *M. tuberculosis* detection rate was higher in milk samples (2.80%; CI: 1.13–5.68) than in blood samples (1.60%; CI: 0.44–4.05) from animals, but this difference was not statistically significant (*p* > 0.05). Geographically, the prevalence of *M. tuberculosis* was more in Dowarabazar (4.00%; CI: 0.10–20.35) (Table 4). The detection rates in other upazilas were estimated as Sylhet Sadar (3.33%; CI: 0.69–9.43), Sunamganj Sadar (3.33%; CI: 0.08–17.22), Jagannathpur (2.22%; CI: 0.06–11.77), and Jaintapur (1.67%; CI: 0.04–4.94). Statistically, we did not find any significant variation (*p* > 0.05) in the occurrence of *M. tuberculosis* among the studied areas (Table 4).

### 3.3. Comparison Between ELISA and PCR

**Figure 3:**
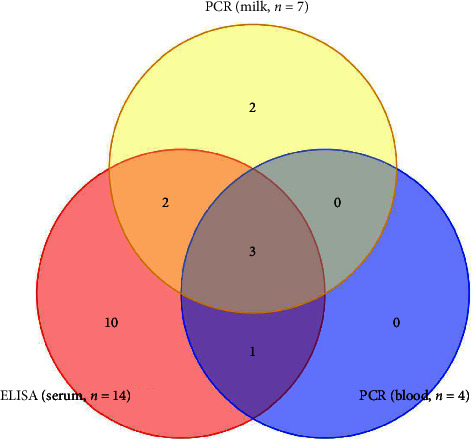
Venn diagram illustrates the overlap of positive detections for *Mycobacterium tuberculosis* in cattle across different sample types using PCR and ELISA.

Results from the ELISA and PCR tests for 250 sera were correlated (*p* < 0.016; Table 4). The univariate analysis revealed that different factors reflect the occurrence of TB in dairy cattle, although we did not find statistically significant variation among all the variables. The Venn diagram highlights the intersections of positive cases in serum, milk, and blood, showcasing the concurrence of results between the two diagnostic methods for a comprehensive assessment of TB presence in the studied population of dairy cattle (Figure 3). Upon creating the Venn diagram, it was observed that 3 animals tested positive across all types of samples—serum, milk, and blood—in both PCR and ELISA. All four blood samples that tested positive through PCR were also found positive in ELISA. Furthermore, positivity in both milk (via PCR) and serum (via ELISA) was observed in two cows. Two cows were singly positive in milk (via PCR), while 10 cows were singly positive in serum (via ELISA) but tested negative in PCR, as shown in Figure 3.

## 4. Discussion

The current literature on bTB in Bangladesh emphasizes the significance of serological investigations in determining the prevalence of the disease and its associated risk factors [9]. Additionally, the detection of *M. tuberculosis* in dairy cattle highlights a potential public health risk, especially in regions with high human TB prevalence. Our findings are consistent with reports from other countries where zoonotic transmission has been documented. The higher prevalence in free-grazing cattle suggests environmental factors may play a role in transmission. Moreover, instances of zoonotic transmission of *M. tuberculosis* among individuals with occupational exposure to cattle in Bangladesh have been recorded [10]. Future studies should focus on longitudinal surveillance and the implementation of One Health strategies to mitigate the risk of zoonotic TB transmission.

In the Sylhet division of Bangladesh, there is a dearth of research on the epidemiology of *M. tuberculosis* infection in cattle populations. Therefore, this study was conducted to get a more comprehensive understanding of the epidemiology of TB in dairy cattle in this region. There have been reports of cases of bovine-origin TB in humans for a long time, and this has had a disastrous effect on both public health and the world economy [13–15]. In this current investigation, we report on crossbred dairy cattle infections of *M. tuberculosis* from commercial and individual dairy farms in the Sylhet division of Bangladesh. This study, the first of its kind in the region, utilized a serological assay for assessing and a PCR to directly diagnose *M. tuberculosis* in biological samples of bovine blood and milk. Serological assays have been shown to be an accurate tool for assessing TB prevalence in European wild animals [16]. The serological test, ELISA, is valuable for screening animal diseases due to its speed, simplicity of use, and affordability [17, 18]. The present study found that around 6.0% (on an average 5.60%) of dairy cattle were seropositive for *M. tuberculosis* in the Sylhet division of Bangladesh, while other studies have reported higher seroprevalence rates, such as 13.82% in India [19], 50% in Egypt, and 51.92% in Iraq [20]. Additionally, the country-wise prevalence of MTBC using ELISA also varied, with 0.87% observed in India [21] and 2% in Ethiopia [22]. Other bovine species have also shown variability in MTBC seroprevalence. For instance, the seroprevalence rate in yaks is 2.2% [23], which is lower than our results. On the other hand, buffaloes showed higher seroprevalence of 10%–11% [24, 25]. Another study conducted on wild boars revealed a significantly higher seroprevalence (17.0%) [26], which was much higher than our findings. The lower seroprevalence rate observed in our study could be attributed to various factors, although it may also be connected to the earlier report of a very low prevalence of bTB in cattle in the Sylhet district of Bangladesh [27].

*M. tuberculosis* infection in cattle has also been previously reported by other investigators from different countries [8, 12]. In this study, we have identified *M. tuberculosis* from blood and milk samples of dairy cattle by PCR as 1.60% and 2.80%, respectively. This finding aligns with previous investigations reporting *M. tuberculosis* infection in cattle from various regions. Research has shown that bTB is prevalent in cattle, leading to significant morbidity and mortality in animals and humans [28]. Cattle are thought to be accidental hosts of *M. tuberculosis*, whereas humans are considered the primary host [29]. It is widely known that MTBC organisms, mainly *M. tuberculosis*, can spread from humans to animals in many different parts of the world. *M. tuberculosis* has been confirmed in tissues and fluids from cattle [3, 17, 30–38] as well as other animals, such as dogs [39], nonhuman primates [40], elephants [41, 42], and parrots [43, 44]. The majority of cases of *M. tuberculosis* in cattle are found in countries with extremely high rates of human infection, suggesting that the disease was introduced into the cattle populations from humans [8]. Bangladesh is one of the top 30 countries with a high burden of TB, accounting for 3.6% of the global total. The estimated incidence and mortality rates of TB in Bangladesh are 221 cases and 24 deaths per 100,000 people, respectively [45]. Despite the problem of human *M. tuberculosis* infections in our country, the prevalence of infection in cattle remains unknown since no prevalence studies have been published to date. According to published reports, cattle contract *M. tuberculosis* primarily from humans, with respiratory transmission being the mode of transmission [46]. *M. tuberculosis* has been found in cattle in a number of different countries, even though there is not always evidence of direct human-to-cattle transmission. Nevertheless, global reports of *M. tuberculosis* transmission from humans to cattle have increased, likely due to higher incidence of the disease in humans than cattle populations [8]. To better understand the role of livestock, particularly cattle, in the human-to-cattle transmission of *M. tuberculosis*, more investigations are needed.

## 5. Conclusions

The results presented in this report clearly demonstrate that *M. tuberculosis* is a significant concern for both veterinary and public health in our country. It also highlights the potential for zoonotic transmission. The report reaffirms the necessity of coordinated efforts between the public health and veterinary agencies to ensure that comprehensive epidemiological investigations are conducted in such situations. A multisectoral One Health approach, involving business, public health, and animal health sectors, is crucial for gaining a better understanding of the epidemiology and implementing preventive measures to protect both human health and animal health.

## Figures and Tables

**Table 1:** Seroprevalence of *Mycobacterium tuberculosis* in dairy cattle from different farms in Sylhet.

Location	*n*/*N*	Prevalence % (95% CI)	Fisher's exact test valuea	*p* value
Intensive commercial dairy farm			6.79	0.58
Farm 1	3/40	7.50% (1.57–20.39)		
Farm 2	1/20	5.00% (0.13–24.87)		
Farm 3	0/20	0.00% (0.00–16.84)∗		
Farm 4	2/20	10.00% (1.24–31.70)		
Farm 5	1/10	10.00% (0.25–44.50)		
Farm 6	0/20	0.00% (0.00–16.84)∗		
Farm 7	1/20	5.00% (0.13–24.87)		
Free-grazing cattle				
Sunamganj Sadar	2/30	6.67% (0.82–22.07)		
Jagannathpur	1/45	2.22% (0.06–11.77)		
Dowarabazar	3/25	12.00% (2.55–31.22)		
Total (seropositive)	14/250	5.60% (3.09–9.22)		
Total (serosuspected)	5/250	2.00% (0.65–4.61)		

*Note:n* = positive case, *N* = total sample.

Abbreviation: CI = confidence interval.

^∗^Superscript means one-sided 97.5% confidence interval; ^a^Superscript means 7 cells (43.8%) have expected count less than 5.

**Table 2:** Molecular detection of *Mycobacterium tuberculosis* from blood of dairy cattle from different farms in Sylhet.

Farm name and location	*n*/*N*	Prevalence % (95% CI)	Fisher's exact test value^a^	*p* value
Intensive commercial dairy farm			5.29	0.93
Farm 1	1/40	2.50% (0.06–13.16)		
Farm 2	1/20	5.00% (0.13–24.87)		
Farm 3	0/20	0.00% (0.00–16.84)∗		
Farm 4	0/20	0.00% (0.00–16.84)∗		
Farm 5	0/10	0.00% (0.00–16.84)∗		
Farm 6	0/20	0.00% (0.00–16.84)∗		
Farm 7	0/20	0.00% (0.00–16.84)∗		
Free-grazing cattle				
Sunamganj Sadar	0/30	0.00%(0.00–16.84)∗		
Jagannathpur	1/45	2.22% (0.06–11.77)		
Dowarabazar	1/25	4.00% (0.10–20.35)		
Total PCR positive in blood	4/250	1.60% (0.44–4.05)		

*Note:n* = positive case, *N* = total sample.

Abbreviation: CI = confidence interval.

^∗^Superscript means one-sided 97.5% confidence interval; ^a^Superscript means 7 cells (43.8%) have expected count less than 5.

**Table 3:** Molecular detection of *Mycobacterium tuberculosis* from milk of dairy cattle from different farms in Sylhet.

Farm name and location	*n*/*N*	Prevalence % (95% CI)	Fisher's exact test value^a^	*p* value
Intensive commercial dairy farm			5.78	0.72
Farm 1	2/40	5.00% (0.61–16.92)		
Farm 2	1/20	5.00% (0.13–24.87)		
Farm 3	0/20	0.00% (0.00–16.84)∗		
Farm 4	0/20	0.00% (0.00–16.84)∗		
Farm 5	0/10	0.00% (0.00–16.84)∗		
Farm 6	0/20	0.00% (0.00–16.84)∗		
Farm 7	1/20	5.00% (0.13–24.87)		
Free-grazing cattle				
Sunamganj Sadar	0/30	0.00% (0.00–16.84)∗		
Jagannathpur	1/45	2.22% (0.06–11.77)		
Dowarabazar	2/25	8.00% (0.98–26.03)		
Total PCR positive in milk	7/250	2.80% (1.13–5.68)		

*Note:n* = positive case, *N* = total sample.

Abbreviation: CI = confidence interval.

^∗^Superscript means one-sided 97.5% confidence interval; ^a^Superscript means 7 cells (43.8%) have expected count less than 5.

**Table 4:** Univariate analysis of different explanatory variables associated with molecular and serological detection of *Mycobacterium tuberculosis* from cattle in Sylhet.

Explanatory variable	*n*/*N*	Prevalence % (95% CI)	Odds ratio (95% CI)	*p* value
Source				
Intensive commercial dairy farm	8/150	5.53% (2.33–10.24)	Ref.	0.82
Free-grazing animals	6/100	6.00% (2.23–12.60)	1.13 (0.38–3.37)	
Diagnostic methods				
PCR	4/250	1.60% (0.44–4.05)	Ref.	0.016
ELISA	14/250	5.60% (3.09–9.22)	3.50 (1.17–10.49)	
Sample type				
Blood	4/250	1.60% (0.44–4.05)	Ref.	0.36
Milk	7/250	2.80% (1.13–5.68)	1.77 (0.51–6.13)	
Location				
Sylhet Sadar	3/90	3.33% (0.69–9.43)	Ref.	0.964
Jaintapur	1/60	1.67% (0.04–4.94)	0.49 (0.05–4.84)	
Sunamganj Sadar	1/30	3.33% (0.08–17.22)	1.00 (0.10–9.99)	
Jagannathpur	1/45	2.22% (0.06–11.77)	0.66 (0.07–6.52)	
Dowarabazar	1/25	4.00% (0.10–20.35)	1.21 (0.12–12.15)	

*Note:n* = positive case, *N* = total sample tested, Ref = means reference category.

Abbreviation: CI = confidence interval.

## Data Availability

The data that support the findings of this study are available from the corresponding author upon reasonable request.

## References

[1] Sa’idu A. S., Okolocha E. C., Dzikwi A. A. (2015). Detection of Mycobacterium Bovis in Organs of Slaughtered Cattle by DNA-Based Polymerase Chain Reaction and Ziehl-Neelsen Techniques in Bauchi State, Nigeria. *Journal of Veterinary Medicine A*.

[2] Mathema B., Kurepina N. E., Bifani P. J., Kreiswirth B. N. (2006). Molecular Epidemiology of Tuberculosis: Current Insights. *Clinical Microbiology Reviews*.

[3] Gumi B., Schelling E., Berg S. (2012). Zoonotic Transmission of Tuberculosis Between Pastoralists and Their Livestock in South-East Ethiopia. *EcoHealth*.

[4] Une Y., Mori T. (2007). Tuberculosis as a Zoonosis From a Veterinary Perspective. *Comparative Immunology, Microbiology and Infectious Diseases*.

[5] Ayele W. Y., Neill S. D., Zinsstag J., Weiss M. G., Pavlik I. (2004). Bovine Tuberculosis: An Old Disease but a New Threat to Africa. *International Journal of Tuberculosis & Lung Disease*.

[6] Cadmus S., Palmer S., Okker M. (2006). Molecular Analysis of Human and Bovine Tubercle Bacilli From a Local Setting in Nigeria. *Journal of Clinical Microbiology*.

[7] Gilbert M., Mitchell A., Bourn D., Mawdsley J., Clifton-Hadley R., Wint W. (2005). Cattle Movements and Bovine Tuberculosis in Great Britain. *Nature*.

[8] Hossai̇n M. B., Sayeed M. A., Faruk M. S. A., Khan M. M., Rumi̇ M. A., Hoque M. A. (2023). Sero-Epidemiology of Bovine Tuberculosis in Dairy Cattle in Chattogram, Bangladesh. *Turkish Journal of Veterinary Research*.

[9] Sarkar S., Haider N., Islam A. (2023). Occurrence of Tuberculosis Among People Exposed to Cattle in Bangladesh. *Veterinary Medicine and Science*.

[10] Prasad C. B., Vise E., Dandapat P. (2022). Laboratory and Field Evaluation of Polymerase Chain Reaction Assays for Diagnosis of Zoonotic Tuberculosis in Bovine Post Mortem Samples. *Indian Journal of Animal Sciences*.

[11] Lombard J. E., Patton E. A., Gibbons-Burgener S. N. (2021). Human-to-Cattle *Mycobacterium tuberculosis* Complex Transmission in the United States. *Frontiers in Veterinary Science*.

[12] Hlokwe T. M., Said H., Gcebe N. (2017). *Mycobacterium tuberculosis* Infection in Cattle From the Eastern Cape Province of South Africa. *BMC Veterinary Research*.

[13] Brett J. L., Humble M. W. (1991). Incidence of Human Tuberculosis Caused by Mycobacterium Bovis. *New Zealand Medical Journal*.

[14] de Kantor I. N., Ritacco V. (1994). Bovine Tuberculosis in Latin America and the Caribbean: Current Status, Control and Eridication Programs. *Veterinary Microbiology*.

[15] Fanning A., Edwards S. (1991). Mycobacterium Bovis Infection in Human Beings in Contact With Elk (*Cervus elaphus*) in Alberta, Canada. *The Lancet*.

[16] Roos E. O., Olea-Popelka F., Buss P. (2018). Seroprevalence of Mycobacterium Bovis Infection in Warthogs (Phacochoerus Africanus) in Bovine Tuberculosis-Endemic Regions of South Africa. *Transboundary and Emerging Diseases*.

[17] Che’ Amat A., González-Barrio D., Ortiz J. A. (2015). Testing Eurasian Wild Boar Piglets for Serum Antibodies Against Mycobacterium Bovis. *Preventive Veterinary Medicine*.

[18] Pérez de Val B., Napp S., Velarde R. (2017). Serological Follow-Up of Tuberculosis in a Wild Boar Population in Contact With Infected Cattle. *Transboundary and Emerging Diseases*.

[19] Prakash C., Kumar P., Joseph B. (2015). Evaluation of Different Diagnostics Tests for Detection of Tuberculosis in Cattle. *Indian Journal of Veterinary Pathology*.

[20] Sci S. B.-A.-A (2012). The Incidence of Bovine Tuberculosis & Its Public Health Hazards in a Dairy Cattle Station in Iraq. *Iraqi Academic Scientific Journals*.

[21] Didugu H., Rn R., Sagi N. R. C. (2016). Seroprevalence of Bovine Tuberculosis in Krishna District of Andhra Pradesh , India. *Research Notes*.

[22] Gumi B., Schelling E., Firdessa R. (2011). Prevalence of Bovine Tuberculosis in Pastoral Cattle Herds in the Oromia Region, Southern Ethiopia. *Tropical Animal Health and Production*.

[23] Gong Q. L., Chen Y., Tian T. (2021). Prevalence of Bovine Tuberculosis in Dairy Cattle in china during 2010–2019: A Systematic Review and Meta-Analysis. *PLoS Neglected Tropical Diseases*.

[24] Sangeet L. (2010). *Prevalence and Risk Factor Assessment of Bovine Tuberculosis: Impact of the Disease in Wildlife, Domestic Animals and Human Interface at Koshi Tappu Wildlife Reserve in Eastern Nepal*.

[25] Khan I. A., Khan A., Mubarak A., Ali S. (2008). Factors Affecting Prevalence of Bovine Tuberculosis in Nili Ravi Buffaloes. *Pakistan Veterinary Journal*.

[26] Varela-Castro L., Alvarez V., Sevilla I. A., Barral M. (2020). Risk Factors Associated to a High *Mycobacterium tuberculosis* Complex Seroprevalence in Wild Boar (*Sus scrofa*) From a Low Bovine Tuberculosis Prevalence Area. *PLoS One*.

[27] Mandal P. K., Ahsan M. I., Apu H. D., Akter S., Ahmed S. S. U., Paul S. (2023). Very Low Prevalence of Bovine Tuberculosis in Cattle in Sylhet District of Bangladesh. *Heliyon*.

[28] Elagdar A., Edris A., Moustafa S., Heikal G. (2022). Prevalence of Tuberculosis in Bovine Slaughtered Animals and Suspected Patients in Gharbia Governorate. *Benha Veterinary Medical Journal*.

[29] Thoen C. O., Steele J. H., Gilsdorf M. J. (2008). *Mycobacterium Bovis Infection in Animals and Humans*.

[30] Fritsche A., Engel R., Buhl D., Zellweger J. P. (2004). Mycobacterium Bovis Tuberculosis: From Animal to Man and Back. *International Journal of Tuberculosis & Lung Disease*.

[31] Ocepek M., Pate M., Žolnir-Dovč M., Poljak M. (2005). Transmission of *Mycobacterium tuberculosis* From Human to Cattle. *Journal of Clinical Microbiology*.

[32] Prasad H. K., Singhal A., Mishra A. (2005). Bovine Tuberculosis in India: Potential Basis for Zoonosis. *Tuberculosis*.

[33] Chen Y., Chao Y., Deng Q. (2009). Potential Challenges to the Stop TB Plan for Humans in China; Cattle Maintain *M. bovis* and *M. tuberculosis*. *Tuberculosis*.

[34] Romero B., Rodríguez S., Bezos J. (2011). Humans as Source of *Mycobacterium tuberculosis* Infection in Cattle, Spain. *Emerging Infectious Diseases*.

[35] Ameni G., Tadesse K., Hailu E. (2013). Transmission of *Mycobacterium tuberculosis* Between Farmers and Cattle in Central Ethiopia. *PLoS One*.

[36] Mittal M., Chakravarti S., Sharma V., Sanjeeth B. S., Churamani C. P., Kanwar N. S. (2014). Evidence of Presence of *Mycobacterium tuberculosis* in Bovine Tissue Samples by Multiplex PCR: Possible Relevance to Reverse Zoonosis. *Transbound. Emerg. Dis.*.

[37] Adesokan H. K., Akinseye V. O., Streicher E. M., Van Helden P., Warren R. M., Cadmus S. I. (2019). Reverse Zoonotic Tuberculosis Transmission From an Emerging Uganda I Strain between Pastoralists and Cattle in South-Eastern Nigeria. *BMC Veterinary Research*.

[38] Malama S., Munyeme M., Mwanza S., Muma J. B. (2014). Isolation and Characterization of Non Tuberculous Mycobacteria From Humans and Animals in Namwala District of Zambia. *BMC Research Notes*.

[39] Parsons S. D. C., Warren R. M., Ottenhoff T. H. M., Gey van Pittius N. C., van Helden P. D. (2012). Detection of *Mycobacterium tuberculosis* Infection in Dogs in a High-Risk Setting. *Research in Veterinary Science*.

[40] Shipley S. T., Coksaygan T., Johnson D. K., Mcleod C. G., Detolla L. J. (2008). Diagnosis and Prevention of Dissemination of Tuberculosis in a Recently Imported Rhesus Macaque (*Macaca mulatta*). *Journal of Medical Primatology*.

[41] Mikota S. K., Peddie L., Peddie J. (2001). Epidemiology and Diagnosis of *Mycobacterium tuberculosis* in Captive Asian Elephants (*Elephas maximus*). *Journal of Zoo and Wildlife Medicine*.

[42] Payeur J. B., Jarnagin J. L., Marquardt J. G., Whipple D. L. (2002). Mycobacterial Isolations in Captive Elephants in the United States. *Annals of the New York Academy of Sciences*.

[43] Hoop R. K. (2002). *Mycobacterium tuberculosis* Infection in a Canary (*Serinus canaria* L.) and a Blue-Fronted Amazon Parrot (*Amazona amazona* Aestiva). *Avian Diseases*.

[44] Schmidt V., Schneider S., Schlömer J., Krautwald-Junghanns M. E., Richter E. (2008). Transmission of Tuberculosis Between Men and Pet Birds: A Case Report. *Avian Pathology*.

[45] Who (2020). *Global Tuberculosis Report*.

[46] Lefford M. J. (1986). Biology of the Mycobacteria, Immunological and Environmental Aspects. *Tubercle*.

